# Clinical outcomes of right ventricular outflow tract stenting compared to surgical shunting in late-presenting children

**DOI:** 10.3389/fcvm.2024.1395132

**Published:** 2024-10-24

**Authors:** Radityo Prakoso, Resi Citra Dewi, Brian Mendel, Celly Anantaria Atmadikoesoemah, Salomo Purba, Damba Dwisepto Aulia Sakti, Nanda Iryuza, Yovi Kurniawati, Renan Sukmawan

**Affiliations:** ^1^ Division of Pediatric Cardiology and Congenital Heart Disease, Department of Cardiology and Vascular Medicine, National Cardiovascular Centre Harapan Kita, Universitas Indonesia, Jakarta, Indonesia; ^2^ Department of Cardiology and Vascular Medicine, National Cardiovascular Centre Harapan Kita, Universitas Indonesia, Jakarta, Indonesia; ^3^ Department of Cardiology and Vascular Medicine, Sultan Sulaiman Government Hospital, North Sumatera, Indonesia; ^4^ Division of Non-Invasive Diagnostic and Cardiovacular Imaging, Department of Cardiology and Vascular Medicine, National Cardiovascular Centre Harapan Kita, Universitas Indonesia, Jakarta, Indonesia; ^5^ Division of Pediatric and Congenital Heart Surgery, Department of Surgery, National Cardiovascular Centre of Harapan Kita, Universitas Indonesia, Jakarta, Indonesia; ^6^ Division of Invasive Cardiology and Non-Surgical Intervention, Department of Cardiology and Vascular Medicine, National Cardiovascular Centre Harapan Kita, Universitas Indonesia, Jakarta, Indonesia

**Keywords:** developing country, fallot tetralogy, late-presenter, modified Blalock-Taussig shunt, RVOT obstruction, stent

## Abstract

**Background:**

Right ventricular outflow tract (RVOT) stenting as an alternative palliation for patients with stenotic RVOTs is increasingly recognized. However, a notable gap remains in the literature regarding the efficacy and the comparative outcomes between RVOT stenting and the modified Blalock-Taussig shunt (mBTS) in children older than one year.

**Methods:**

We conducted a retrospective review of clinical data from patients aged one year to 18 years with stenotic RVOTs who underwent RVOT stenting or mBTS procedures at our institution between December 2019 and October 2022. We compared major adverse cardiovascular events (MACE) including re-hospitalization, re-intervention, and mortality, hospital and ICU length of stay, and discharge oxygen saturation between the groups.

**Results:**

We identified 58 patients (51.7% male) with a median age of 2.6 years (IQR: 2–8.1) and a median weight of 9.7 kg (IQR: 7.5–17.5). Among them, 18 (31%) patients received RVOT stenting, and 40 (68.9%) patients had mBTS. The median age for the RVOT stenting group was 92.5 months (IQR: 31.2–152) compared to 26.5 months (IQR: 23.0–54.0) for the mBTS group (*p* = 0.218). MACEs occurred in 4 (22.2%) patients with RVOT stents and 8 (20%) patients with mBTS (*p* = 0.624). Patients with mBTS had a longer ICU stay (median 3.5 days, IQR, 2–5) compared to those with RVOT stents (median 2 days, IQR: 1–2) (*p* = 0.295). Conversely, the hospital stay for patients with mBTS was shorter (median 10 days, IQR, 7.5–13.7) than for those with RVOT stents (median 11.5 days, IQR, 7–19) (*p* = 0.045). The median discharge oxygen saturation was 87% (IQR: 83–88) in the mBTS group and 80% (IQR: 75–87) in the RVOT stenting group (*p* = 0.212).

**Conclusions:**

RVOT stenting as palliation to stenotic RVOTs in children older than one year demonstrated outcomes comparable to mBTS in terms of MACE and achieving oxygen saturation targets.

## 1 Introduction

Stenotic right ventricular outflow tract (RVOT) lesions with ventricular shunts are the most common form of cyanotic congenital heart disease (1). When definitive surgical repair is not feasible, palliative interventions become essential. The modified Blalock-Taussig shunt (mBTS) is often employed to increase pulmonary blood flow (2). However, previous research has reported high rates of morbidity and mortality, as well as variations in the growth and development of pulmonary arteries associated with the mBTS (3, 4). An emerging palliative approach involves non-surgical interventions, such as ductal stenting and RVOT stenting (3, 5, 6). While some studies have demonstrated positive clinical outcomes with RVOT stenting compared to the mBTS, there is limited research specifically examining the role of RVOT stenting in late presenters, particularly those older than one year (7, 8). Some literature suggests that RVOT stenting not only yields favorable outcomes in older children as a bridging therapy but also serves as a rescue procedure for patients at high surgical risk (8). This study, therefore, aims to compare the initial outcomes of RVOT stenting vs. mBTS in patients aged one to eighteen years with RVOT obstruction and ventricular shunts.

## 2 Methods

### 2.1 Study design and population

We conducted a retrospective review of clinical data for all pediatric patients aged one to 18 years with RVOT obstruction and a ventricular shunt who underwent palliative RVOT stenting or mBTS at our institution between December 2019 and October 2022. Ethical approval was granted by the Institutional Review Board of the National Cardiovascular Center Harapan Kita (No. LB.02.01/VII/035/KEP035/2022). The study included patients with congenital heart lesions such as Tetralogy of Fallot (TOF), double outlet right ventricle (DORV) with ventricular septal defect (VSD), and pulmonary stenosis (PS) with VSD. Patients who had undergone single-stage complete repair for TOF or who had single-ventricle physiology (e.g., tricuspid atresia, VSD) were excluded. An anomalous coronary artery course was not considered a contraindication for either RVOT stenting or mBTS. The need for palliative treatment was determined by institutionally recognized risk factors that precluded definitive repair surgery, such as low weight, small pulmonary arteries, and other significant comorbidities. The decision to perform RVOT stenting was primarily influenced by severe comorbidities that posed a high risk for mBTS. Key indications for RVOT stenting included: (1) profound desaturation (typically below 40%–50%), (2) low ejection fraction (LVEF < 40%), (3) high risk associated with performing an mBTS, and (4) high risk for total repair despite adequate pulmonary artery size. All procedures were performed without cardiopulmonary bypass, either electively or emergently, based on clinical judgment.

Patient characteristics before palliation, including age, gender, nutritional status, diagnosis, oxygen saturation, type and average size of the stent or shunt, type of operation, left ventricular ejection fraction (LVEF), and McGoon ratio, were recorded. Intra-procedural and post-procedural data, such as oxygen saturation and complications (e.g., bleeding, stent or shunt failure, sudden cardiac arrest), were also documented. Follow-up assessments were conducted, with all data retrieved from electronic medical records (EMR).

### 2.2 Study outcome

The primary outcomes focused on major adverse cardiac events (MACE), including rehospitalization and re-intervention. Secondary outcomes included discharge oxygen saturation, length of stay (LOS) in the intensive care unit (ICU), and total hospital LOS.

### 2.3 Statistical analyses

Categorical data were presented as frequencies (*n*) and percentages (%). Numerical data were expressed as median and interquartile range (IQR). Chi-square or Fisher's exact tests were used to compare categorical data, while independent Mann-Whitney tests were employed for numerical data analysis. Spearman correlation coefficients were used to evaluate associations between different outcomes. Statistical analyses were performed using the Statistical Package for Social Science (SPSS) version 26.0, with significance set at *p* < 0.05.

## 3 Results

### 3.1 Patient demographics

We identified 58 patients (51.7% male) with a median age of 2.6 years and a median weight of 9.7 kg between December 2019 and October 2022. Of these, 18 patients (31%) received RVOT stenting, while 40 patients (68.9%) had mBTS. Patients in the RVOT stenting group were older, with a median age of 92.5 months compared to 26.5 months for the mBTS group (*p* = 0.218).

Table 1 shows the baseline characteristics of the groups. There were no significant differences in age, gender, body weight, or nutritional status. Tetralogy of Fallot was the most common diagnosis in both groups. Emergency procedures were more frequent in the RVOT stent group (77.8%) compared to the mBTS group (32.5%). The pre-procedural McGoon ratio was slightly higher in the RVOT stent group (*p* = 0.745). The median stent size was 9 mm, while the median shunt size was smaller at 4 mm (*p* = 0.874).

**Table 1:** Patient demographics and clinical characteristics underwent RVOT stent implantation and mBTS procedure.

Variable	*N* (overall%)	RVOT stent (*N* = 18)	mBTS (*N* = 40)	*P* value
Sex				0.158
Female, *n* (%)	28 (48.2)	9 (50)	19 (47.5)	
Male, *n* (%)	32 (55.1)	9 (50)	21 (52.5)	
Age, month, median (IQR)	32 (24–101)	92.5 (31.2–152)	26.5 (23–54)	0.218
Body weight, kg, median (IQR)	9.7 (7.5–18)	16.5 (7.6–32)	9 (7.5–34)	0.104
Nutritional status				0.085
Normal, *n* (%)	14 (24.1)	7 (38.8)	7 (17.5)	
Low, *n* (%)	27 (46.5)	9 (50)	18 (45)	
Very low, *n* (%)	17 (29.3)	2 (11.1)	15 (37.5)	
Diagnosis				0.361
TOF, *n* (%)	48 (82.7)	15 (83.3)	33 (82.5)	
DORV VSD, PS, *n* (%)	8 (13.7)	2 (11.1)	6 (15)	
VSD PS, *n* (%)	2 (3.4)	1 (5.6)	1 (2.5)	
Anomalous coronary artery course, *n* (%)	3 (5.1)	0 (0)	3 (7.5)	0.200
Procedural status				0.676
Elective, *n* (%)	31 (53.4)	4 (22.2)	27 (67.5)	
Emergency, *n* (%)	27 (46.5)	14 (77.8)	13 (32.5)	
Left ventricular ejection fraction/LVEF,%, median (IQR)	71 (64–76)	69 (31–74)	72 (65–77)	0.213
Oxygen saturation pre-procedure,%, median (IQR)	69.5 (65–75)	69 (53–72)	69.5 (65.7–74)	0.150
McGoon ratio, median (IQR)	1.3 (1.2–1.5)	1.3 (1.2–1.5)	1.2 (1.1–1.5)	0.745
Stent or shunt diameter, mm, median (IQR)	5 (4–9)	9 (8–10)	4 (4–5)	0.874
Stent length, mm, median (IQR)		38 (29–39)		
Type of operation (*n*)			Median sternotomy (15), thoracotomy (25)	
Stent type (*n*)		Omnilink Elite^TM^ (Abbott, United States) (11), Dynamic^TM^ (Biotronik, Germany) (7)		

RVOT, right ventricular outflow tract; mBTS, modified Blalock Taussig shunt; TOF, Tetralogy of Fallot; DORV, double outlet right ventricle; VSD, ventricular septal defect; PS, pulmonary stenosis; IQR, interquartile range.

### 3.2 Procedural complications

Several complications occurred during and after the procedures (see Table 2). In the RVOT stenting group, one stent dislodged (5.6%) and required surgical intervention. One patient (11.1%) had cardiac arrest during balloon dilatation, but this was resolved. In the mBTS group, bleeding was controlled during surgery without postoperative issues. Overall, complication rates during the procedures did not differ significantly (*p* = 0.075).

**Table 2:** Procedural complications of RVOT stent and mBTS during and post-procedure.

Variable	*N* (overall%)	RVOT stent (*N* = 18)	mBTS (*N* = 40)	*P* value
During procedure complications, *n* (%)	5 (8.6)	3 (16.7)	2 (5)	0.075
Bleeding, *n* (%)	1 (1.7)	0 (0)	1 (2.5)	0.398
Stent/shunt failure, *n* (%)	1 (1.7)	1 (5.6)	0 (0)	0.540
Sudden cardiac arrest, *n* (%)	2 (3.4)	1 (11.1)	1 (2.5)	0.721
Post procedure complications, *n* (%)	19 (32.7)	4 (22.2)	15 (37.5)	0.054
Stent/shunt failure, *n* (%)	5 (8.6)	1 (5.6)	4 (10)	0.396
Infection, *n* (%)	10 (17.2)	1 (5.6)	9 (22.5)	0.757
Bleeding, *n* (%)	2 (3.4)	0 (0)	2 (5)	0.593

RVOT, right ventricular outflow tract; mBTS, modified Blalock – Taussig shunt.

During hospitalization, one patient (5.6%) in the RVOT stenting group experienced restenosis, while shunt failure occurred in 10% of patients in the mBTS group, requiring re-intervention. Bleeding complications were more frequent in the mBTS group, with 5% requiring clot evacuation. Infection rates were higher in the mBTS group at 22.5% (*p* = 0.593). Major adverse cardiovascular event (MACE) probability was higher in the mBTS group compared to the RVOT stent group (*p* = 0.704).

### 3.3 Clinical outcomes

The primary clinical outcome, which included rehospitalization (*p* = 0.512), re-intervention (*p* = 0.174), and mortality (*p* = 0.869), did not differ significantly between the groups (see Table 3). Major adverse cardiac events (MACEs) were slightly higher in the RVOT stenting group, occurring in 22.2% of patients, compared to 20% in the mBTS group (*p* = 0.624). Rehospitalization due to desaturation occurred in 5.6% of patients in the RVOT stent group. Re-intervention rates were 11.1% in the RVOT stent group and 15% in the mBTS group (*p* = 0.174). Mortality was observed in one patient (5.6%) in the RVOT stent group due to infection and in two patients (5.0%) in the mBTS group due to shunt thrombosis and low cardiac output, all during hospitalization.

**Table 3:** Clinical outcome of RVOT stent implantation and mBTS.

Variable	*N* (overall%)	RVOT stent (*N* = 18)	mBTS (*N* = 40)	*P* value
MACE	12 (20.6)	4 (22.2)	8 (20)	0.624
Re-hospitalization, *n* (%)	1 (1.7)	1 (5.6)	0 (0)	0.512
Re-intervention, *n* (%)	8 (13.7)	2 (11.1)	6 (15)	0.174
Mortality, *n* (%)	3 (5.1)	1 (5.6)	2 (5)	0.869
Achieved oxygen saturation target, *N* (%)	58 (100)	18 (100)	40 (100)	0452
Discharged oxygen saturation,%, median (IQR)	86 (79–88)	80 (75–87)	87 (83–88)	0212
ICU LOS, days, median (IQR)	3 (2–6)	2 (1–2)	3.5 (2–5)	0.295
Total hospital LOS, days, median (IQR)	10 (7–15)	11.5 (7–19)	10 (7.5–13.7)	0.045
Total repair, *n* (%)	24 (41.3)	9 (50)	15 (37)	0.342
Time from palliation to total repair, months, median (IQR)	3.5 (3–6)	3 (3–5)	4 (3–6)	0.289
Duration of follow-up, months, median (IQR)	33 (27.7–41.5)	30.5 (26.5–32.0)	38.5 (28.7–47)	0.111

RVOT, right ventricular outflow tract; mBTS, modified Blalock – Taussig shunt; LOS, length of stay; IQR, interquartile range.

The hospital stay was longer in the RVOT stenting group, with a median of 11.5 days compared to 10 days in the mBTS group (*p* < 0.05). The median ICU stay for the RVOT stenting group was 2 days, compared to 3.5 days for the mBTS group (*p* = 0.295). Median discharge oxygen saturation was 80% in the RVOT stenting group and 87% in the mBTS group (*p* = 0.212). Total repair was performed in 50% of the RVOT stent group and 37.5% of the mBTS group. The duration from palliation to total repair was 3 months in the RVOT stent group and 4 months in the mBTS group, with no statistically significant difference between the two (*p* = 0.289).

## 4 Discussions

### 4.1 Late presenters’ characteristics

Our study population predominantly comprised late presenters (7–9), a stark contrast to previous studies that focused on younger age groups (10–12). This discrepancy is primarily due to diagnostic and management limitations, as well as a shortage of pediatric cardiologists and cardiac surgeons. Most of our patients were diagnosed with Tetralogy of Fallot (TOF), and their older age at presentation mirrors findings from studies in regions with limited healthcare access. In Indonesia, 86.2% of patients with cyanotic congenital heart disease (CHD) were diagnosed late, with TOF being the most common diagnosis (13). Late presenters often exhibit hypertrophy of the infundibulum, making implanted stents more prone to fracture (7–9).

### 4.2 RVOT stenting advantages

RVOT stenting offers several advantages for patients with Tetralogy of Fallot (TOF), particularly those who present late (7–9). In our study, RVOT stenting was not associated with a significant increase in major adverse cardiac events (MACE), with rates similar to those observed in the mBTS group. A key benefit of RVOT stenting was the shorter length of stay in both the ICU and hospital overall. Patients in the RVOT stent group had a significantly shorter median ICU stay, and total hospital stays were also reduced compared to those in the mBTS group. Additionally, discharge oxygen saturation levels were adequate in the RVOT stent group, indicating that the procedure effectively improved pulmonary blood flow without negatively affecting clinical outcomes (10–12).

These findings align with previous studies, such as those by Abumehdi et al. (14) and Quandt et al. (5), which reported similar reductions in ICU length of stay following RVOT stenting. Abumehdi et al. (14) reported a median ICU stay of 2 days, while Quandt et al. (5) found a median stay of 0 days, with only 22% of RVOT stent patients requiring post-procedural ICU admission. RVOT stent implantation directly channels blood flow from the right ventricle into the pulmonary circulation, improving oxygenation without compromising aortic diastolic perfusion pressure. This contributes to enhanced hemodynamic stability, leading to shorter ICU stays and overall reduced hospitalization times (10–12).

### 4.3 RVOT stenting technical difficulties

While RVOT stenting offers several advantages, it also presents significant technical challenges (15). In our study, complications such as stent dislodgement and cardiac arrest during balloon dilation were observed, highlighting the critical need for technical proficiency and meticulous procedural planning. Although mortality rates were comparable between the RVOT stenting and mBTS groups, the RVOT stent patients were often in more severe preoperative conditions, which may have contributed to postoperative complications and mortality. Importantly, however, no deaths in our cohort were directly attributable to the RVOT stenting procedure.

Currently, no stents are specifically designed or approved for use in pediatric patients (16). Nevertheless, stent implantation has emerged as a viable short-term solution for infants and small children with native or post-surgical branch pulmonary artery stenosis. Despite this, certain challenges persist due to the small vessel size, limited vascular access, and the ongoing growth of pediatric patients, which can result in restenosis at the stent site. Intimal hyperplasia is a common complication following stent placement (16).

An ideal stent for pediatric use would need to be crimped onto small balloons, have a low-profile delivery system, offer sufficient radial strength, resist intimal hyperplasia, and have the capacity to be expanded to adult dimensions while maintaining structural integrity. Haddad et al. (2023) demonstrated the safe implantation and efficacy of 3 Optimus-L stents (AndraTec, Koblenz, Germany) using a low-profile approach in small patients (16).

In our study, the presence of an anomalous coronary artery course was not considered a contraindication to RVOT stenting, despite the inherent risks. A key limitation of the procedure is the close proximity of coronary branches to the anticipated landing zone of the pulmonary valve, which raises concerns about coronary compression due to the radial force exerted by the balloon-expandable stent. This risk is particularly significant for patients with the left anterior descending (LAD) artery originating from the right aortic sinus, as the LAD crosses near the RVOT (17). However, Hanser et al. (17) (2022) assessed outcomes of RVOT stenting in TOF patients with anomalous coronary courses, finding no mortality in their cohort despite the presence of these anomalies. Out of 122 TOF patients, 10 had coronary anomalies, with 6 requiring reintervention and 1 undergoing early surgical palliation due to suboptimal stent placement. Their findings indicate that RVOT stenting remains a safe and effective option in such cases (17).

### 4.4 Expected outcomes

According to Martins et al. (18), the ideal timing for definitive corrective surgery during the first year of life is typically between 3 and 6 months for asymptomatic or mildly symptomatic children. However, our study population differs significantly in terms of age, with many patients presenting late (7–9). For patients who receive RVOT stents, the long-term management strategy often involves a staged approach to definitive repair, with the timing guided by the size of the pulmonary arteries, typically assessed using a McGoon ratio of 1.5 or higher. In some instances, mBTS or RVOT stenting may still be performed even when the McGoon ratio exceeds 1.5 to ensure sufficient pulmonary artery development. Patients with RVOT stents usually remain clinically stable until they are ready for total repair (19, 20). Our findings further support RVOT stenting as a safe and effective bridge to definitive surgical correction in late-presenting TOF patients, facilitating better planning for surgery once the patient is in a more favorable clinical condition. The limited number of completed repairs can be attributed to patients feeling clinically improved and thus declining further procedures. This issue is exacerbated by the complex and lengthy queuing system within the healthcare framework, leading to extended wait times at national referral centers. Despite these challenges, we strive to maintain follow-up through clinic visits and direct communication with patients.

## 5 Limitations

This investigation relied on retrospective data sourced from registries or medical records, necessitating meticulous attention to the accuracy and comprehensiveness of the records. Patient selection was consecutive, with individuals not randomized into intervention groups but rather chosen based on clinical and surgical risk evaluations. Notably, the sample size within the RVOT stent group remains constrained.

## 6 Conclusions

RVOT stenting as a palliation to stenotic RVOTs in children showed comparable outcomes to mBTS in terms of MACE and achievement of oxygen saturation targets in patients older than one year.

## Data Availability

The original contributions presented in the study are included in the article/Supplementary Material, further inquiries can be directed to the corresponding author.
